# Diabetes mellitus in patients with heart failure and reduced ejection fraction: a post hoc analysis from the WARCEF trial

**DOI:** 10.1007/s11739-024-03544-4

**Published:** 2024-02-23

**Authors:** Giulio Francesco Romiti, Katarzyna Nabrdalik, Bernadette Corica, Tommaso Bucci, Marco Proietti, Min Qian, Yineng Chen, John L. P. Thompson, Shunichi Homma, Gregory Y. H. Lip, the WARCEF Investigators

**Affiliations:** 1 Liverpool Centre for Cardiovascular Science at University of Liverpool, Liverpool John Moores University and Liverpool Heart & Chest Hospital, Liverpool, UK; 2 Department of Translational and Precision Medicine, Sapienza – University of Rome, Rome, Italy; 3 Department of Internal Medicine, Diabetology and Nephrology, Faculty of Medical Sciences in Zabrze, Medical University of Silesia, Katowice, Poland; 4 Department of General Surgery and Surgical Specialties “Paride Stefanini”, Sapienza University of Rome, Rome, Italy; 5 Department of Clinical Sciences and Community Health, University of Milan, Milan, Italy; 6 Division of Subacute Care, IRCCS Istituti Clinici Scientifici Maugeri, Milan, Italy; 7 Mailman School of Public Health, Columbia University, New York, USA; 8 Center for Preventive Ophthalmology and Biostatistics, University of Pennsylvania, Philadelphia, Pennsylvania, USA; 9 Cardiology Division, Columbia University Medical Center, New York, USA; 10 Danish Center for Health Services Research, Department of Clinical Medicine, Aalborg University, Aalborg, Denmark

**Keywords:** Diabetes mellitus, Heart failure, HFrEF, Outcomes, Prognosis

## Abstract

Patients with heart failure with reduced ejection fraction (HFrEF) and diabetes mellitus (DM) have an increased risk of adverse events, including thromboembolism. In this analysis, we aimed to explore the association between DM and HFrEF using data from the “Warfarin versus Aspirin in Reduced Cardiac Ejection Fraction” (WARCEF) trial. We analyzed factors associated with DM using multiple logistic regression models and evaluated the effect of DM on long-term prognosis, through adjusted Cox regressions. The primary outcome was the composite of all-cause death, ischemic stroke, or intracerebral hemorrhage; we explored individual components as the secondary outcomes and the interaction between treatment (warfarin or aspirin) and DM on the risk of the primary outcome, stratified by relevant characteristics. Of 2294 patients (mean age 60.8 (SD 11.3) years, 19.9% females) included in this analysis, 722 (31.5%) had DM. On logistic regression, cardiovascular comorbidities, symptoms and ethnicity were associated with DM at baseline, while age and body mass index showed a nonlinear association. Patients with DM had a higher risk of the primary composite outcome (Hazard Ratio [HR] and 95% Confidence Intervals [CI]: 1.48 [1.24–1.77]), as well as all-cause death (HR [95%CI]: 1.52 [1.25–1.84]). As in prior analyses, no statistically significant interaction was observed between DM and effect of Warfarin on the risk of the primary outcome, in any of the subgroups explored. In conclusion, we found that DM is common in HFrEF patients, and is associated with other cardiovascular comorbidities and risk factors, and with a worse prognosis.

**Supplementary Information:**

The online version contains supplementary material available at 10.1007/s11739-024-03544-4.

## Introduction

Currently, approximately 530 million adults worldwide are living with diabetes mellitus (DM), translating to 10% of the general adult population [1]. Heart failure (HF), however, affects up to 64 million people worldwide, with prevalence ranging between 1 and 3%. Incidences of both DM and HF are also rising [2, 3], and as a result, DM and HF are often found together. Indeed, DM can foster the onset of HF [4]: epidemiological trends show that up to 30% of patients with HF also have DM, with figures higher in hospitalized patients, and increasing over the last decades [5, 6].

The pathophysiology underlying the relationship between DM and HF is complex and only partially understood [7, 8]. DM promotes the onset and progression of HF through atherosclerosis, ischemic heart disease, and loss of myocardial function [8, 9]; hyperglycemia itself has detrimental effects on the myocardium [10]. Furthermore, DM can induce other risk factors for HF (or enhance their effects), including arterial hypertension, atherogenic dyslipidemia, thrombogenesis, and inflammation [8].

Among the detrimental effects of HF, the promotion of a hypercoagulable state has been repeatedly described [11]. This contributes to the higher risk of ischemic stroke which is found in patients with HF [12], even in the absence of other known causes of thromboembolic risk, such as atrial fibrillation (AF) [13]. DM bolsters thromboembolic risk [14] and has been described as a potential factor that defines a subgroup of patients with HF and reduced ejection fraction (HFrEF) that may be at particularly higher risk of stroke [15]. Hence, it would be anticipated that different antithrombotic drugs may have different effects in the “high-risk” DM subgroup.

The Warfarin versus Aspirin in Reduced Cardiac Ejection Fraction (WARCEF) trial compared warfarin vs. aspirin in patients with HFrEF and sinus rhythm and found no significant differences in the primary composite outcome of ischemic stroke, intracerebral hemorrhage, or death from any cause [16]. A previous comprehensive analysis of WARCEF subgroups showed that the effect of treatment did not differ in patients with and without DM, both before and after adjustment for multiple covariates [17]. Beyond this, however, the effects of DM in this context remain unclear.

In this additional post hoc analysis of the WARCEF trial, our primary aim was to analyze the association between DM and prognosis of patients with HFrEF. We also explore whether there may be a different effect of warfarin vs. aspirin in some subgroups of DM patients.

## Methods

Full details on the design, follow-up, and primary results of the WARCEF trial were previously reported [16, 18]. Briefly, the trial was conducted between October 2002 and January 2010 and enrolled 2305 patients with HFrEF. Patients eligible for inclusion were adults (≥ 18 years) with HFrEF (left ventricular ejection fraction ≤ 35% assessed by echocardiography, or radionuclide or contrast ventriculography within 3 months before randomization), normal sinus rhythm, and no contraindication to receive warfarin therapy; those with a clear indication for either warfarin or aspirin were not eligible for inclusion. Moreover, while patients in any functional class of the New York Heart Association (NYHA) classification could be included, patients in NYHA class I could account for ≤ 20% of the total sample size. Other eligibility criteria included a modified Rankin score ≤ 4 and planned treatment with beta-blockers, angiotensin-converting enzyme inhibitor (or angiotensin receptor blocker where indicated), or hydralazine and nitrates [16]. The main exclusion criteria were conditions associated with a high risk of cardiac embolism, such as atrial fibrillation (AF), mechanical heart valve, endocarditis, or intracardiac mobile or pedunculated thrombus. Follow-up was performed with an initial planned maximum duration of 5 years, further extended to 6 years; the trial’s primary outcome was the composite of ischemic stroke (IS), intracerebral hemorrhage (ICH), or death from any cause. The study adhered to the principles of the Declaration of Helsinki; the study protocol was approved by the international review boards and ethics boards of participating centers, and written informed consent was provided by all patients. The trial was registered at ClinicalTrials.gov (NCT00041938).

For each patient, baseline information on medical history and comorbidities, including the presence of DM, was collected using the customized Web-based WARCEF data management system. For this analysis, we included all patients with data available on the presence of DM at baseline.

### Study outcomes

Full details on the outcome definition and adjudication in WARCEF are reported elsewhere [16, 18]. The aim of the WARCEF trial was to compare warfarin vs. aspirin, on a primary composite outcome of ischemic stroke, intracerebral hemorrhage, or death from any cause, analyzed in a time-to-first event fashion. In this post hoc analysis, we aimed to evaluate the association between DM and prognosis of patients with HFrEF, on the primary outcome as defined in the WARCEF trial. We also evaluated other exploratory secondary outcomes (i.e., the individual components of the primary composite outcome: all-cause death, IS, or ICH) and also explored if there was a different effect of warfarin vs. aspirin in some subgroups of patients with DM.

### Statistical analysis

Continuous variables were expressed as mean and standard deviation (SD), and differences were evaluated using Student’s *t*-test. Categorical variables were reported as counts and percentages, and differences were evaluated using the chi-square test.

To evaluate factors associated with DM at baseline, we performed a multiple logistic regression analysis. Covariates included were age and Body Mass Index (BMI) (both modeled as restricted cubic splines with 4 knots, with age = 65 years and BMI = 25 kg/m^2^ as references), sex, smoking habit (current vs. ex/never), race or ethnic group, NYHA class (I–II vs. III–IV), and history of hypertension, stroke/transient ischemic attack (TIA), myocardial infarction (MI), peripheral vascular disease (PVD), and atrial fibrillation (AF). Results were reported as adjusted odds ratio (aOR) and corresponding 95% confidence intervals (CI) for categorical variables; the relationship between continuous variables and aOR and 95% CI for the presence of DM was reported graphically.

For both primary and exploratory secondary outcomes, incidence rates (IR) and corresponding 95% CI were reported, according to the presence of DM. To analyze the association between history of DM and the risk of outcomes, we used multiple adjusted Cox regression models. Covariates included were age and BMI (both modeled as restricted cubic splines with 4 knots), sex, treatment allocation (warfarin or aspirin), smoking habit (current vs. ex/never), race or ethnic group, NYHA class (I–II vs. III–IV), and history of hypertension, stroke/TIA, MI, PVD, and AF. Results were reported as adjusted hazard ratio (aHR) and corresponding 95% CI.

Additionally, we evaluated the effect of DM on the primary composite outcome in relevant subgroups of patients (i.e., according to age, sex, NYHA class, race/ethnicity, smoking status, history of hypertension, stroke/TIA, MI, PVD, and AF); we also explored if the effect of the study drugs (i.e., warfarin vs. aspirin) on the risk of the primary composite outcome was different in patients with vs. without DM, through an interaction analysis, stratified by clinical relevant characteristics (age, sex, NYHA class, history of hypertension, stroke/TIA, MI, and PVD).

A two-sided *p* < 0.05 was considered statistically significant. All analyses were performed using R 4.2.3 (R Core Team, Vienna, Austria) for Windows.

## Results

Among 2305 patients originally enrolled in the WARCEF trial, 2294 (99.5%, mean age 60.8 (11.3) years, 19.9% females) had available data on the presence of DM at baseline and were included in this analysis. Of these, 722 (31.5%) had DM.

Baseline characteristics according to the presence of DM are reported in Table 1. Patients with DM were older (62.5 (9.8) years vs 60.0 (11.9) years, *p* < 0.001) and had higher BMI (30.8 (6.2) vs 28.4 (5.7) kg/m^2^, *p* < 0.001); they also showed a higher prevalence of non-Hispanic Black and other ethnicities and most comorbidities, including hypertension, MI, and history of stroke/TIA. Patients with DM also showed worse symptoms and lower rates of current smoking or alcohol use.

**Table 1:** Baseline characteristics according to the presence of diabetes Mellitus at baseline

Variable, *n*/total (%)	No diabetes mellitus (*n* = 1572)	Diabetes mellitus (*n* = 722)	*P*
***Demographics***			
Age, mean (SD)	60.0 (11.9)	62.5 (9.8)	< 0.001
*Continent*			< 0.001
North America	698/1572 (44.4)	414/722 (57.3)	
Europe	805/1572 (51.2)	285/722 (39.5)	
Latin America	69/1572 (4.4)	23/722 (3.2)	
Female sex	310/1572 (19.7)	147/722 (20.4)	0.764
*Race or ethnic group*			0.028
Non-Hispanic White	1210/1572 (77.0)	520/722 (72.0)	
Non-Hispanic Black	214/1572 (13.6)	116/722 (16.1)	
Hispanic	110/1572 (7.0)	56/722 ( 7.8)	
Other	38/1572 (2.4)	30/722 ( 4.2)	
***Physical examination***			
BMI, kg/m^2^, mean (SD)	28.4 (5.7)	30.8 (6.2)	< 0.001
Systolic blood pressure, mmHg, mean (SD)	123 (19)	126 (19)	< 0.001
Diastolic blood pressure, mmHg, mean (SD)	75 (12)	73 (11)	0.017
Heart rate, bpm, mean (SD)	71.5 (12.0)	73.0 (11.8)	0.006
***Medical history***			
Hypertension	847/1530 (55.4)	517/698 (74.1)	< 0.001
Atrial fibrillation	51/1572 ( 3.2)	35/722 ( 4.8)	0.079
Myocardial infarction	695/1571 (44.2)	416/722 (57.6)	< 0.001
Ischemic cardiomyopathy	616/1571 (39.2)	375/722 (51.9)	< 0.001
Peripheral vascular disease	142/1572 (9.0)	119/722 (16.5)	< 0.001
History of stroke/TIA	177/1572 (11.3)	117/721 (16.2)	0.001
*Smoking status*			< 0.001
Current	313/1571 (19.9)	94/720 (13.1)	
Ex	784/1571 (49.9)	394/720 (54.7)	
Never	474/1571 (30.2)	232/720 (32.2)	
*Alcohol consumption*			0.002
Current	424/1572 (27.0)	147/721 (20.4)	
Ex	327/1572 (20.8)	179/721 (24.8)	
Never	821/1572 (52.2)	395/721 (54.8)	
*NYHA class*			< 0.001
NYHA I	228/1566 (14.6)	87/720 (12.1)	< 0.001
NYHA II	910/1566 (58.1)	353/720 (49.0)	
NYHA III	412/1566 (26.3)	268/720 (37.2)	
NYHA IV	16/1566 (1.0)	12/720 (1.7)	
Left ventricular ejection fraction, %, mean (SD)	25.0 (7.6)	25.0 (7.2)	0.990
***Treatments***			
Randomized to warfarin	767/1572 (48.8)	371/722 (51.4)	0.267
Aspirin^†^	842/1462 (57.6)	400/655 (61.1)	0.146
Warfarin^†^	118/1572 (7.5)	61/722 (8.4)	0.485
ACE inhibitor/ARB	1546/1568 (98.6)	708/722 (98.1)	0.437
Beta-blocker	1402/1569 (89.4)	657/722 (91.0)	0.256
Aldosterone blocker	546/912 (59.9)	265/431 (61.5)	0.613
Nitrate	332/1569 (21.2)	211/721 (29.3)	< 0.001
Calcium channel blocker	108/1567 (6.9)	95/721 (13.2)	< 0.001
Diuretic	1240/1569 (79.0)	612/722 (84.8)	0.001
Statin	870/1097 (79.3)	521/578 (90.1)	< 0.001
Device therapy			
Pacemaker	182/1572 (11.6)	103/722 (14.3)	0.081
Implantable cardioverter defibrillator	276/1572 (17.6)	142/722 (19.7)	0.247

*ACE*: angiotensin-converting enzyme, *ARB*: angiotensin receptor blocker, *BMI:* Body Mass Index, *NYHA:* New York Heart Association functional class, *SD:* standard deviation

† Data are for the use of these medications before the patients underwent randomization

**Fig. 1:**
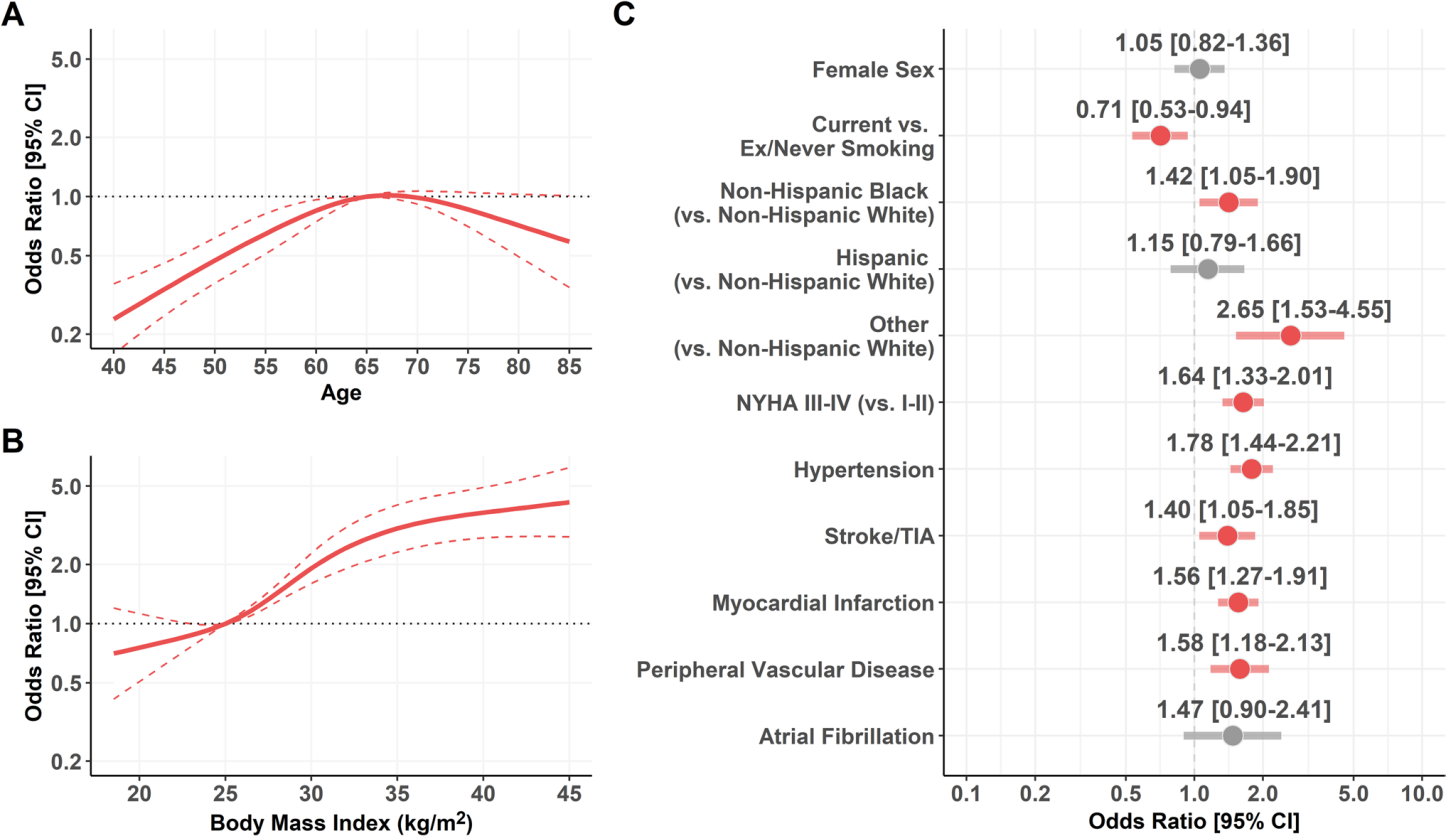
Association between baseline characteristics and diagnosis of diabetes mellitus at baseline. Panel **A:** age (*p* for nonlinearity < 0.001); Panel **B:** Body Mass Index (p for nonlinearity = 0.009); and Panel **C:** other categorical variables. *CI* : confidence interval, *NYHA*: New York Heart Association, *TIA:* transient ischemic attack

When we analyzed factors associated with a diagnosis of DM at baseline, age and BMI were nonlinearly associated with odds of DM (Fig. 1; panel A and B; p for nonlinearity < 0.001 and 0.009, respectively). Specifically, odds of DM decreased with age below and above 65; conversely, the likelihood of DM increased sharply for BMI between 25 and 30 kg/m^2^, reaching a plateau thereafter. We also found that non-Hispanic Black (OR [95% CI]: 1.42 [1.05–1.90]) and other ethnicities (OR [95% CI]: 2.65 [1.53–4.55]) had higher odds of DM, when compared to non-Hispanic White patients. Moreover, higher NYHA classes (OR [95% CI]: 1.64 [1.33–2.01]) and history of hypertension (OR [95% CI]: 1.78 [1.44–2.21]), stroke/TIA (OR [95% CI]: 1.40 [1.05–1.85]), MI (OR [95% CI]: 1.56 [1.27–1.91]), and PVD (OR [95% CI]: 1.58 [1.18–2.13]) were all associated with higher odds of diagnosis of DM at baseline. Conversely, current smoking status showed an inverse association (Fig. 1, panel C).

**Fig. 2:**
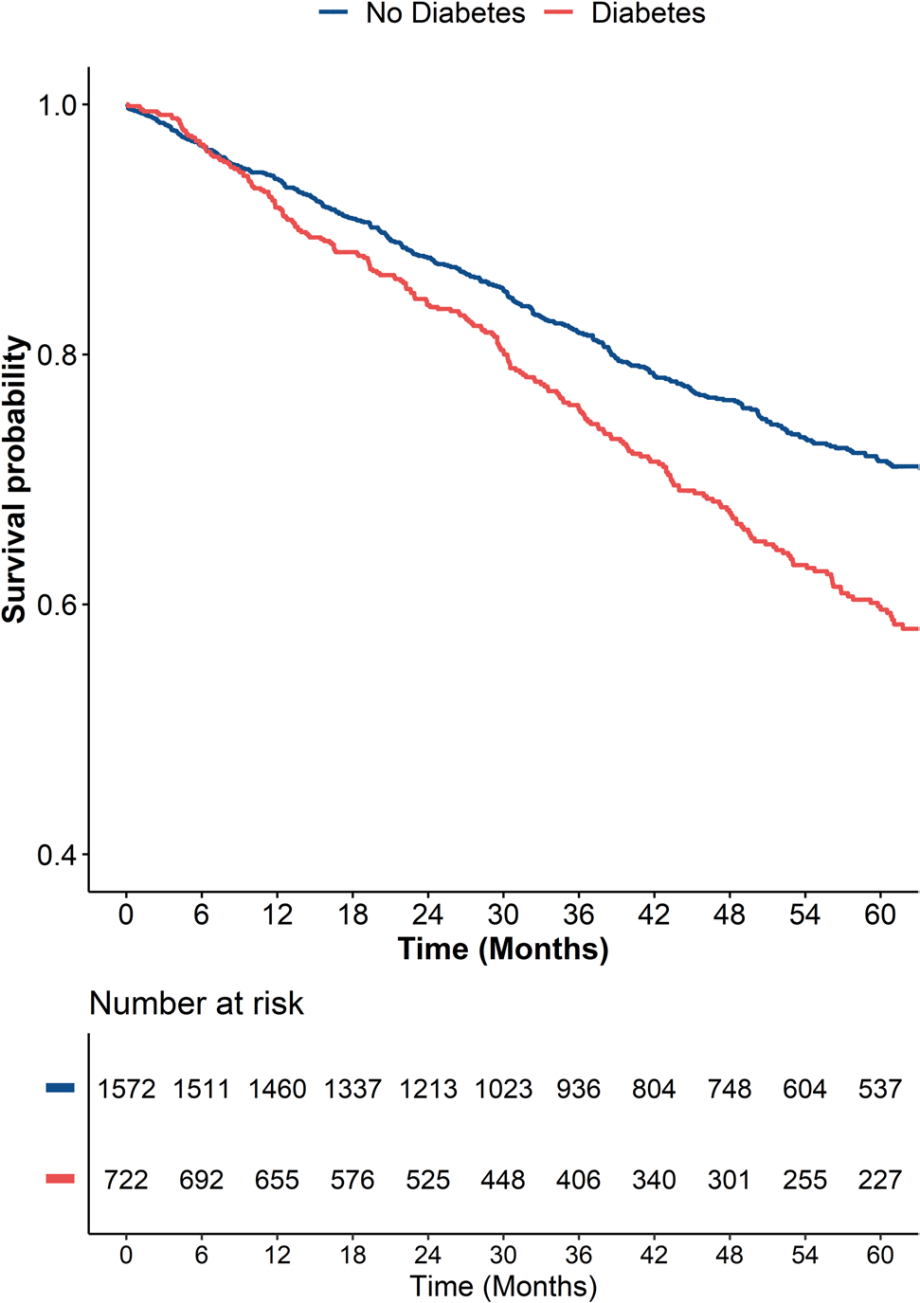
Kaplan–Meier curves for the primary composite outcome according to the presence of diabetes mellitus at baseline. Log-rank *p* < 0.001

After a median follow-up of 3.4 years (Interquartile range 2.0–5.0 years), patients with DM showed a higher incidence of the primary composite outcome (IR [95% CI]: 10.0 [8.7–11.3] per 100 person-years) compared to patients without DM (IR [95% CI]: 6.7 [6.0–7.4] per 100 person-years). Similar results were observed for all-cause death (IR [95% CI]: 8.6 [7.4–9.8] and 5.7 [5.1–6.3] per 100 person-years for patients with and without DM, respectively) and IS (IR [95% CI]: 1.3 [0.9–1.8] and 0.9 [0.7–1.2] per 100 person-years for patients with and without DM, respectively), while incidence for ICH was similarly low in patients with and without DM (Table 2). Results of the Kaplan–Meier curves for the primary composite outcome (Fig. 2) showed lower survival probability in patients with DM (log-rank *p* < 0.001).

**Table 2:** Event count and incidence rates for the risk of the primary and secondary outcomes according to the diagnosis of diabetes mellitus

Group	Event count	Events per 100 person-years IR [95%CI]	aHR (95% CI)	*p*
***Primary composite outcome***				
No diabetes Mellitus	374/1572	6.7 [6.0–7.4]	Ref	Ref
Diabetes mellitus	244/722	10.0 [8.7–11.3]	1.48 (1.24–1.77)	** < 0.001**
***Secondary outcomes***				
*All-cause death*				
No diabetes mellitus	317/1572	5.7 [5.1–6.3]	Ref	Ref
Diabetes mellitus	210/722	8.6 [7.4–9.8]	1.52 (1.25–1.84)	** < 0.001**
*Ischemic stroke*				
No diabetes mellitus	53/1572	0.9 [0.7–1.2]	Ref	Ref
Diabetes mellitus	31/722	1.3 [0.9–1.8]	1.19 (0.72–1.96)	0.508
*Intracerebral hemorrhage*				
No diabetes mellitus	4/1572	0.1 [0.0–0.2]	Ref	Ref
Diabetes mellitus	3/722	0.1 [0.0–0.4]	2.71 (0.47–15.50)	0.262

*aHR:* adjusted hazard ratio,* CI:* confidence interval, *IR:* Incidence Rate

Results of the Cox regression analyses for the risk of primary and exploratory secondary outcomes are reported in Table 2. DM was associated with a higher hazard of the primary composite outcome (aHR [95% CI]: 1.48 [1.24–1.77]) and all-cause mortality (aHR [95% CI]: 1.52 [1.25–1.84]). No statistically significant differences were observed for the risk of IS and ICH.

Subgroup analyses for the risk of the primary outcome are reported in Fig. S1 in Supplementary Materials. No statistically significant interaction was observed for any of the characteristics explored and the risk of all-cause death, IS, and ICH in patients with vs. without DM. Nonetheless, some evidence for a higher magnitude of DM effect was observed in patients aged 75 or more (*p*_int_ = 0.158) and in females (*p*_int_ = 0.105).

Finally, when we analyzed the interaction between DM and the effect of randomized treatment on the risk of the primary composite outcome, stratified by relevant subgroups, we confirmed the previous finding [17] of no statistically significant interaction (Fig. S2 in Supplementary Materials).

## Discussion

In this post hoc ancillary study of the WARCEF trial, our principal findings are as follows: (1) DM was common in patients with HFrEF and was associated with other risk factors, including higher BMI, symptoms, and cardiovascular comorbidities, and (2) DM was associated with a significantly higher risk of the primary composite outcome during follow-up, with some evidence of higher effect exerted by DM in elderly and females patients. We also confirmed the previous observation that DM was not associated with a significantly different effect of warfarin vs. aspirin on the risk of the primary composite outcome, in any of the subgroups explored, despite the higher risk profile of patients with DM.

The prevalence of DM that we found in our cohort is similar to those observed in other trials performed in patients with HFrEF patients [19, 20]. This confirms that DM is a highly prevalent disease in this clinical setting; moreover, we found some evidence of ethnic differences in the prevalence of DM, with non-White patients being more likely to present with DM at baseline, in line with previous findings [21]. We also showed how DM is associated with more severe symptoms and with a higher burden of cardio-metabolic conditions. Although the cross-sectional nature of our analysis does not allow for inference on whether these results are directly attributable to a DM-specific effect, these results suggest that patients with DM and HFrEF present with a more complex phenotype, in line with the hypothesis that DM can foster the occurrence of HF through several pathways [22].

We also observed an association between DM and the risk of the primary composite outcome; similar results were found when considering death as an individual event. Conversely, only nonsignificant results were observed for the other two components (IS and ICH), perhaps due to the relatively low incidence of these events in this trial. These results appear consistent with previous evidence arising from randomized clinical trials (RCTs) [23] and also from real-world observational studies [24, 25], which showed a detrimental effect of DM on the prognosis of patients with HF. In the subgroup analyses, we found broadly consistent effects of DM on the risk of the primary outcome across relevant subgroups. We, however, found some evidence of a greater detrimental effect of diabetes on the risk of the primary outcome in patients ≥ 75 years and in women: In these subgroups, DM doubled the risk of the primary outcome, although without a statistically significant interaction.

These results expand previous evidence on how DM influences outcomes in patients with HFrEF. Indeed, previous studies already showed how women are disproportionally affected by the detrimental effects of DM on quality of life and outcomes [26]. Our results, although without reaching statistical significance, suggest that some subgroups of HFrEF patients may be more prone to the consequences of the DM-HFrEF interaction. While further evidence is needed to confirm and expand these observations, our analysis provides insights that may be useful in identifying those patients who may need closer surveillance. Indeed, female representation in clinical trials of patients with HF has been repeatedly advocated as a potential area of improvement [27, 28], and sex-based undertreatment [27, 29] could also contribute to these results. Similar considerations may be applied to elderly patients [30, 31].

When we evaluated how DM modified the effect of the randomized treatment on the risk of the primary outcome in relevant subgroups, we found no statistically significant interaction, reproducing the results found in the earlier WARCEF subgroup analysis, which showed no overall interaction between DM at baseline and effect of warfarin vs. aspirin [17], as noted above. Of note, these findings are also consistent with those of the COMMANDER-HF trial, which randomized patients with recent worsening HFrEF, sinus rhythm, and coronary artery disease to receive low-dose rivaroxaban or placebo on top of antiplatelet therapy [32]: while subgroup analyses did not show differences in patients with DM, some evidence of a potential lower effect of anticoagulation in patients with DM was also observed, similar to our analysis [32]. We expanded these previous observations, showing that these results are consistent across a wide range of subgroups, who may present different risks of adverse events. Although our analysis of the interaction by subgroups was limited by the overall low power to detect differences, these results still provide interesting preliminary evidence to foster future research.

Indeed, several hypotheses may explain our findings. Platelet activity is enhanced in patients with DM due to several mechanisms [33, 34], which also include upregulation of Nox2: This has been previously described in patients with DM and linked with an increased risk of cardiovascular events in these subjects [35, 36]. Overall, the role of platelets is currently considered crucial in the pathophysiology of thrombosis in patients with DM [37]. Indeed, although a potential lower efficacy of aspirin has been hypothesized in patients with DM (also due to accelerated platelet turnover [38, 39]), aspirin is still widely used and recommended for the prevention of cardiovascular events in patients with DM and particularly for secondary prevention [37]. The central role of platelets in the pathophysiology of thrombotic events in patients with DM may contribute to explain our findings. Nonetheless, oral anticoagulation may provide some potential advantages, as shown by a post hoc analysis of the COMMANDER-HF trial, in which the use of low-dose anticoagulant and antiplatelets was able to reduce the risk of thromboembolic events, although these were not the main determinants of morbidity and mortality in patients recruited in the trial [40]. While current evidence does not support the implementation of such approaches in clinical practice, future studies may be able to identify subgroups of DM patients who may gain some benefit from more complex antithrombotic strategies.

Taken together, our results have clinical implications. We showed that patients with DM and HFrEF are more complex, more symptomatic, and with a higher burden of cardiovascular diseases compared to patients without DM. This interplay impacts prognosis, an effect which we found driven by all-cause mortality. Of note, antithrombotic treatment received did not influence prognosis in DM-HFrEF patients, despite their high risk of thromboembolic events. This may support the hypothesis that the complexity of patients with DM and HFrEF requires further efforts to improve prognosis and a more comprehensive and holistic management. Our results appear therefore consistent with recent guidelines recommendations, which call for the implementation of multidisciplinary and integrated care approaches in HFrEF patients [41], and with recent evidence which showed how the overall burden of morbidity, frailty, and complexity (encompassing also social determinants of health) represent powerful determinants of adverse outcomes in HF patients [42–44].

### Strength and limitations

We acknowledge some limitations. First, this is a post hoc, non-prespecified analysis of a randomized trial; therefore, we may did not have adequate power to detect differences, especially regarding subgroup and interaction analyses. Results should therefore be interpreted with caution and as hypothesis-generating. Nonetheless, our findings appear consistent with previous evidence and have biological plausibility. WARCEF collected DM status at baseline but not other potentially relevant factors, such as duration of disease and glycemic control; we also did not have data regarding drugs for the treatment of DM. Of note, the WARCEF trial was conducted 20 years ago, when treatment options and recommendations for both DM and HFrEF were different and more limited; therefore, we were unable to explore the effect of potentially interesting drugs (such as sodium-glucose cotransporter-2 inhibitors or glucagon-like peptide-1 receptor agonists) on the relationship between DM and HFrEF. We also acknowledge the risk for other potential unaccounted confounders, which effect we cannot exclude, although we adjusted our regression models to account for the most relevant confounders. Finally, we focused our analysis on the primary composite outcome of all-cause death, IS, and ICH, using a time-to-first event approach, as in the main trial analysis. Our exploratory secondary outcomes were therefore represented by the individual components of the composite endpoint, and we observed low rates for nonfatal events, thus reducing our power to detect differences in patients with and without DM. As these analyses were also not adjusted for multiple comparisons, the results should be interpreted with caution and as hypothesis-generating.

## Conclusions

In the WARCEF trial, DM was found in 1 out of 3 HFrEF patients and was associated with ethnicity, age, BMI, and other cardiovascular comorbidities. Patients with HFrEF and DM showed worse prognosis, with some evidence of a higher effect in women and elderly patients. Patients with DM and HFrEF may require a personalized and holistic approach to improve their prognosis.

### Supplementary Information

Below is the link to the electronic supplementary material.

Supplementary file1 (DOCX 950 KB)

## Data Availability

The data that support the findings of this study are available from the corresponding author upon reasonable request and with the agreement of the WARCEF PIs.
