# Supplementary material for: Diabetes mellitus in patients with heart failure and reduced ejection fraction: a post hoc analysis from the WARCEF trial
Source: Intern Emerg Med. 2024 Feb 23;19(4):931–9. doi: 10.1007/s11739-024-03544-4 (PMC11186946; doi:10.1007/s11739-024-03544-4)

**Diabetes Mellitus in patients with Heart Failure and reduced Ejection Fraction: a post-hoc analysis from the WARCEF Trial**

Giulio Francesco Romiti^1,2^ MD*, Katarzyna Nabrdalik^1,3^ MD* Bernadette Corica^1,2^ MD Tommaso Bucci^1,4^ MD, Marco Proietti^5,6^ MD PhD, Min Qian^7^ PhD, Yineng Chen^8^ MS, John L.P. Thompson^7^ PhD, Shunichi Homma^9^ MD, Gregory Y.H. Lip^1,10^ MD;
the WARCEF Investigators

*Supplementary Materials*

**Figure S1 – Subgroup Analysis for the risk of the Primary Outcome according to the presence of Diabetes Mellitus**

**Legend:** NYHA= New York Heart Association; p_int_=p for interaction


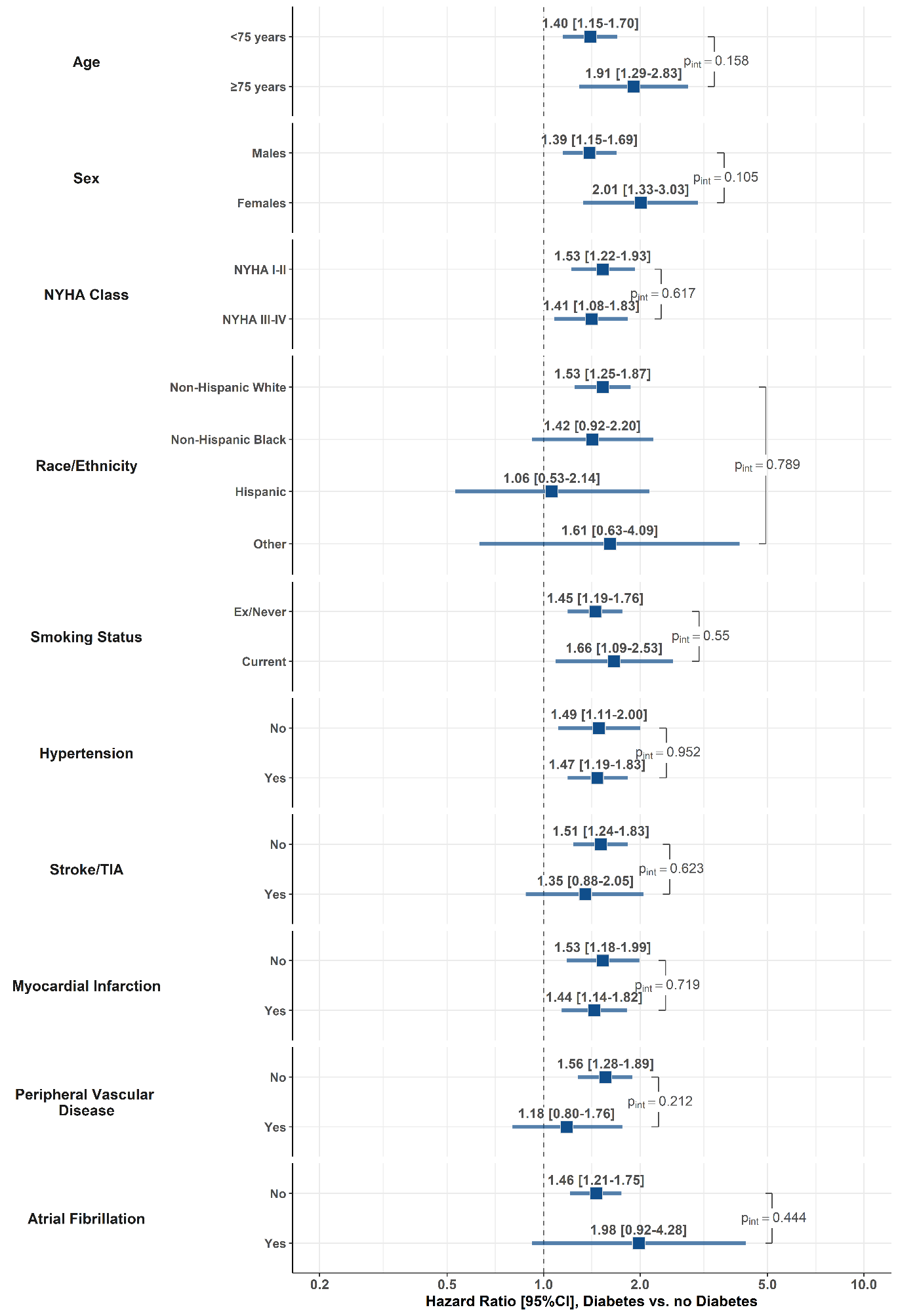


**Figure S2 – Interaction analysis between Diabetes Mellitus and effect of randomized treatment, stratified by relevant baseline characteristics.**

**Legend:** NYHA= New York Heart Association; p_int_=p for interaction


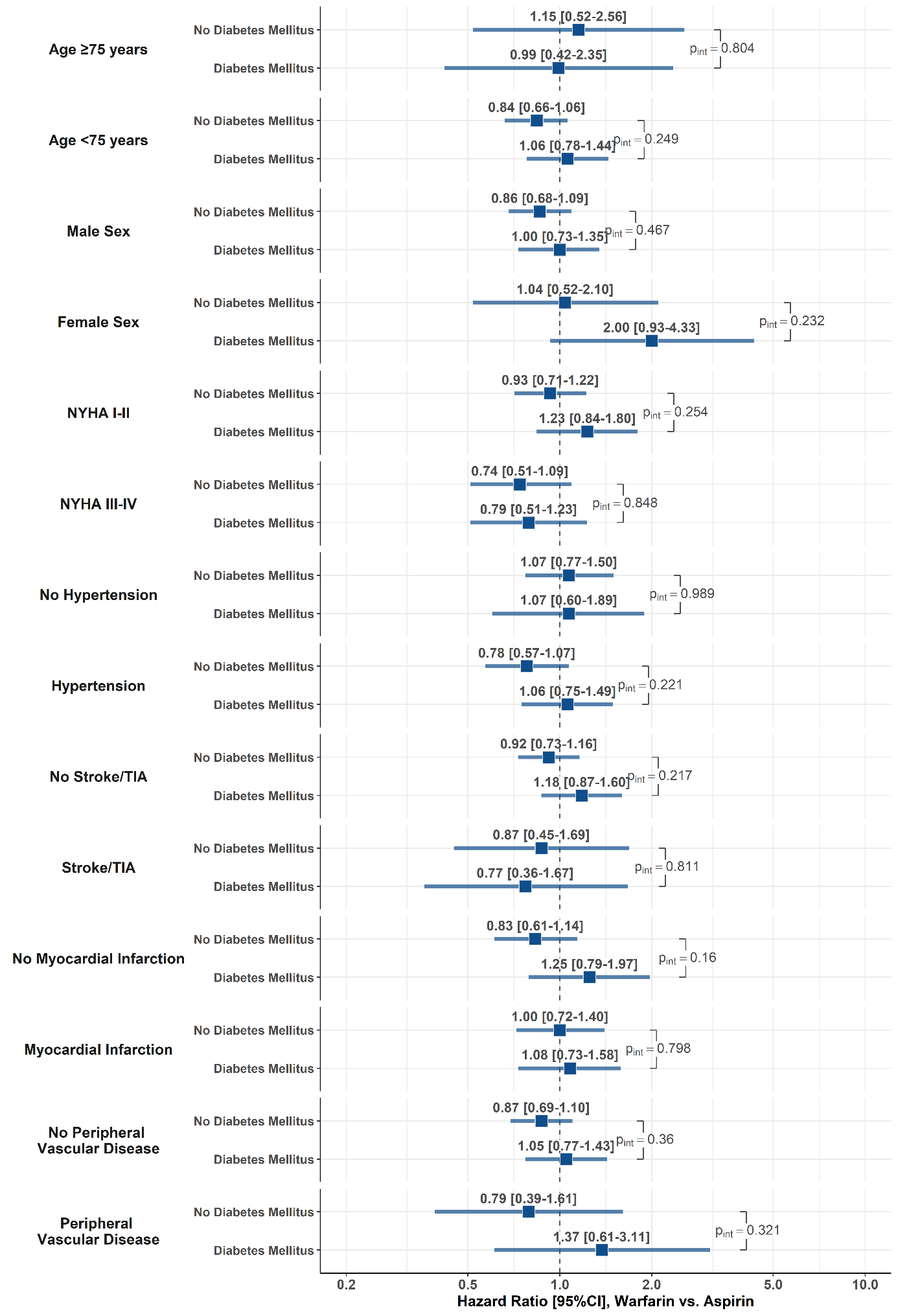

Supplement: Supplementary file 1 — Supplementary file1 (DOCX 950 KB) [file 11739_2024_3544_MOESM1_ESM.docx]
